# A Mobile and Ubiquitous Approach for Supporting Frailty Assessment in Elderly People

**DOI:** 10.2196/jmir.2529

**Published:** 2013-09-04

**Authors:** Jesús Fontecha, Ramon Hervás, José Bravo, Fco Javier Navarro

**Affiliations:** ^1^Esc Sup de InformáticaMAmI Research LabUniversity of Castilla-La ManchaCiudad RealSpain; ^2^Health Service of Castilla-La Mancha, SpainGeriatric ServicesResidencia Asistida de AncianosCiudad RealSpain

**Keywords:** frailty, mobile computing, similarity, elderly people

## Abstract

**Background:**

Frailty is a health condition related to aging and dependence. A reduction in or delay of the frailty state can improve the quality of life of the elderly. However, providing frailty assessments can be difficult because many factors must be taken into account. Usually, measurement of these factors is performed in a noncentralized manner. Additionally, the lack of quantitative methods for analysis makes it impossible for the diagnosis to be as complete or as objective as it should be.

**Objective:**

To develop a centralized mobile system to conduct elderly frailty assessments in an accurate and objective way using mobile phone capabilities.

**Methods:**

The diagnosis of frailty includes two fundamental aspects: the analysis of gait activity as the main predictor of functional disorders, and the study of a set of frailty risk factors from patient records. Thus, our system has several stages including gathering information about gait using accelerometer-enabled mobile devices, collecting values of frailty factors, performing analysis through similarity comparisons with previous data, and displaying the results for frailty on the mobile devices in a formalized way.

**Results:**

We developed a general mechanism to assess the frailty state of a group of elders by using mobile devices as supporting tools. In collaboration with geriatricians, two studies were carried out on a group of 20 elderly patients (10 men and 10 women), previously selected from a nursing home. Frailty risk factors for each patient were collected at three different times over the period of a year. In the first study, data from the group of patients were used to determine the frailty state of a new incoming patient. The results were valuable for determining the degree of frailty of a specific patient in relation to other patients in an elderly population. The most representative similarity degrees were between 73.4% and 71.6% considering 61 frailty factors from 64 patient instances. Additionally, from the provided results, a physician could group the elders by their degree of similarity influencing their care and treatment. In the second study, the same mobile tool was used to analyze the frailty syndrome from a nutritional viewpoint on 10 patients of the initial group during 1 year. Data were acquired at three different times, corresponding to three assessments: initial, spontaneous, and after protein supplementation. The subsequent analysis revealed a general deterioration of the subset of elders from the initial assessment to the spontaneous assessment and also an improvement of biochemical and anthropometric parameters in men and women from the spontaneous assessment to the assessment after the administration of a protein supplement.

**Conclusions:**

The problem of creating a general frailty index is still unsolved. However, in recent years, there has been an increase in the amount of research on this subject. Our studies took advantage of mobile device features (accelerometer sensors, wireless communication capabilities, and processing capacities among others) to develop a new method that achieves an objective assessment of frailty based on similarity results for an elderly population, providing an essential support for physicians.

## Introduction

Resistance and physiological reserves decrease in elderly people, leading to cumulative wear and an increased risk of adverse health effects; this leads to a frailty state. However, frailty is a difficult term to conceptualize and, in most cases, is related to aging, disability, and comorbidity. In the 1970s, this concept was first used to describe a group of elderly people who preserved their independence in a precarious way. Nowadays, there are many definitions and models of frailty. Woodhouse defined frail elderly people as “those greater than 65 years of age who are dependent on other people to perform their basic needs” [1], whereas Gillick (1989) described frail older persons as “old debilitated individuals who cannot survive without the help from others” [2]. More recently, Brocklehurst defined frailty as “the risk of losing the ability to live in the community” [3]. Meanwhile, Buchner and Wagner [4] proposed a definition from a biological point of view. Certainly, the frailty state is composed of multiple domains, as Rockwood describes [5]. Thus, the clinical syndrome of frailty is determined by different symptoms and signs, resulting in the phenotype of frailty proposed by Fried [6]. This author sets out five criteria to determine whether a person is frail or not.

Notably, Hamerman described the difficulty of addressing the concept of frailty due to the large number of parameters to be considered [7]. In fact, detection and diagnosis of frailty must be studied in the following domains: medical, functional, socioeconomic, cognitive, and institutional. The functional domain has been classically appreciated as the independence level of a person. This includes performing activities of daily living [8,9]. In this case, frailty is often equated with functional dependence in these activities, although frail elderly people are sometimes described in predominantly medical terms. However, it is difficult to standardize an operational definition of frailty while taking into account this broad perspective.

Nowadays, the results for frailty detection and diagnosis are based on global scores from standard questionnaires completed by physicians; an overview of the elderly person and his/her environment; measures from medical instruments; and an analysis of lab reports from the elderly patient.

Moreover, doctors do not take into account all the previous items at the final assessment, and their decision is based only on a subset of items. In addition, the first two items depend on the physician’s viewpoint, thereby influencing the final result. For example, the assessments of gait and balance—two of the main indicators for frailty diagnosis—are obtained by several questionnaires. However, current technologies provide mechanisms to obtain the results in a more appropriate manner. For instance, the use of mobile phones with built-in accelerometers as medical instruments during gait and balance activities, in combination with other factors, can successfully generate more accurate and centralized results of frailty, providing much more information than the current tests. In the last decade, many researchers have included new technologies and standardized methods in their works on the frailty syndrome, due to the large number of factors under consideration. For this purpose, Martin presented an overview of the relevant tools (tests and scales) used by researchers in the field of frailty, studying the importance of each tool and the provided information [10]. Jones proposed a method to determine a frailty index from a detailed geriatric assessment focused on studying a set of variables, including balance, communication, cognitive state, nutrition, continence, activities of daily living, and comorbidity among others [11]. However, he mentioned that the best way to measure frailty remains unresolved. In another paper, Rockwood proposed a method based on the results of scales and a statistical study to establish a frailty index related to a specific population [12]. In the same manner, Searle et al proposed a quantification procedure for creating a frailty index from a dataset of variables [13]. In this case, non-numerical variables were coded. Additionally, Gobbens et al defined a conceptual framework to group the most important experimentally detected factors related to frailty [14]. These included cognitive factors, strength, balance, nutrition, physical activity, and mobility, while social and psychological factors were less important.

In recent years, the mobile computing paradigm and the use of mobile devices in health care systems have grown significantly, although integration and deployment remain a challenge [15]. In this paper, we present a system with a corresponding user-friendly mobile application to provide frailty assessments focusing on the analysis of the main parameters of frailty through the study of similarities between individuals, with the aim of supporting frailty decision making and subsequent treatments by doctors and geriatricians.

## Methods

### Overview

In this work, the detection and diagnosis of the frailty state includes two fundamental aspects. The first involves gathering and processing gait information through accelerometer sensors, an important element of frailty detection. The second is the study of all frailty risk factors found in the patient record (including information on gait analysis), providing valid results, and looking at the detection and diagnosis of frailty and pre-frailty in an accurate and objective manner, for interpretation by doctors on their mobile devices, such as mobile phones.

### Identification of Frailty Factors

The identification of frailty factors involves identifying the relevant factors related to frailty. These factors must be included in the system as frailty variables. A set of relevant factors is to be taken into account when a physician conducts a frailty evaluation. Espinoza identified a group of possible risk factors from the frailty phenotype and a systematic review [16]. Additionally, the physical characteristics of frailty are considered; however, the importance of each one is not indicated, at least not in a quantitative manner.

Clinical variables related to frailty come from the patient record as mentioned. The score from tests and scales, the results of lab reports, and general information, among others, are stored by physicians to be studied as needed. Meanwhile, social and psychological indicators are not considered because they do not have a direct relationship with the patient record. The most common indicators are associated with the clinical groups presented in Table 1 [17] (all these items can be quantified easily).

**Figure 1:**
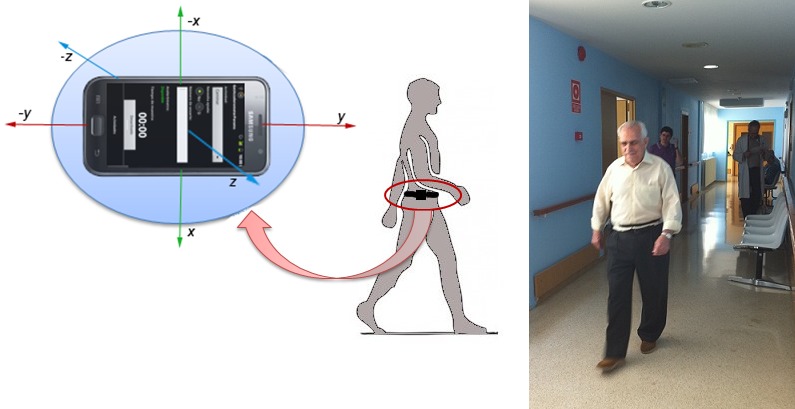
Position of the mobile phone during the performance of the Tinetti gait test and real application.

Functional assessment is the most important domain for determination of frailty and is the first to be studied. For this, the physician applies gait and balance tests to assess several features, mainly based on the Tinetti test [18]. The use of an accelerometer attached to the elder’s waist during these activities collects relevant data on gait and balance. Fontecha et al identified the following indicators from the movement analysis as accelerometry indicators (for the three axes) [19]: arithmetic mean, standard deviation, absolute mean difference, acceleration mean, variance, amplitude, and Pearson coefficient of variation. This new group of parameters is also considered part of the frailty assessment. We propose collecting and analyzing these movement parameters using a mobile phone. (Figure 1 shows the correct position of the mobile device on the elder’s waist.) Apart from these factors, new parameters can be identified to contribute to the final assessment.

**Table 1:** Frailty risk factors from the patient record [17].

Anthropometric and general data	Gender, age, size, weight, Body Mass Index, body mass, lean mass, fat mass, total water, drug number.
Functional assessment	Tinetti gait and balance score, Barthel index, Lawton & Brody score, Get-Up and Go score, need help in physical activities.
Independence in the activities of daily living	Elders can be independent, mild dependent, moderate dependent, great dependent, or serious dependent.
Geriatric syndromes	Checking for dementia, depression, incontinence, immobility, recurrent falls, polypharmacy, comorbidity, sensory deprivation, pressure ulcers, malnutrition, terminal illness.
Nutritional assessment	Total protein, serum albumin, cholesterol level, triglycerides, blood iron, ferritin, vitamin B12, serum folic acid, serum transferrin, leukocytes, lymphocytes, hemoglobin, calcium.
Cognitive assessment	Mini Mental Status score, “Cruz Roja” [17] mental scale.
Pathologies and diseases	Chronic diseases can be divided into several groups: cardiovascular, neurological, respiratory, digestive, endocrine, orthopedic, osteomuscular, eyes, “ear, nose, and throat” disorders, and dermatological.

### The Frailty Diagnosis Model

The system developed is based on a model proposed in a previous work [20], which favors the development of mobile tools for the analysis of the identified frailty risk factors.

This model is divided into two parts: conceptual and functional. The conceptual part defines the set of entities that form the model (ie, which elements are necessary for the frailty assessment). In this case, the mobile phone is the crux of the model and the most important entity. These entities have been grouped into five classes as follows: (1) Devices, referring to the necessary devices in the environment, ie, mobile phone, accelerometer, and server, (2) Users*,* referring to the physician and the elderly patient, (3) Artifacts, which consists of questionnaires and medical instruments that measure aspects related to frailty, (4) Procedures, corresponding to the elements related to the storage of clinical data (from the patient record as well as from the frailty assessment system), and (5) Services, corresponding to the internal services for data acquisition, data processing, data storage, and creation of results.

Additionally, each entity is responsible for one or more actions (known as the entity role). More detailed information about entities and their relationships is given in Multimedia Appendix 1.

Separately, the functional part describes “how” the previous items work according to their roles. Thus, functionalities will be offered by means of a service-oriented approach in which two kinds of services have been identified (mobile and Web services). The first includes the internal services that can be deployed by the mobile device or smartphone. The second is related to services hosted on a server as Web services. For this second kind of service, a network connection (via wireless network) between the mobile device and the server is required.

In a real scenario, the services are run in the order according to Table 2, with inputs and outputs. The inputs represent needed elements or processes to run the services, whereas the outputs are the results provided by the services.

If a patient is studied more than once, this implies the gathering of new values for the frailty variables. Therefore, considering all of the above, we can use a collection of the patient instances rather than a collection of the patients, where an instance consists of a complete set of frailty variables associated with a patient at a given moment, as Fontecha defined [19].

### The Frailty Assessment Process

The main purpose of the tool relates to the frailty assessment, generating more accurate and centralized results for frailty, focusing on the analysis of the main parameters and the application of similarity algorithms. Additionally, we propose the use of hierarchical structures such as treemaps [21] to display the final results on the mobile device. A treemap is a method for displaying hierarchical information in a compact manner using nested colored rectangles, maximizing the available space (in this case, the display of the mobile device), favoring the interpretation of results.

The implementation of the frailty assessment process uses cluster analysis features and similarity algorithms, providing a coefficient for each elder related to a relative frailty assessment in a specific elderly population. This procedure includes three stages. The first is the selection of relevant variables, when the frailty risk factors previously detailed are selected for study. The second is the normalization of variables, which is a necessary procedure because the selected variables may have different types (quantitative, qualitative, or binary) and units. Thus, all identified variables are normalized or standardized before the similarity calculation. This implies that different instances of a variable (eg, weight) must be measured in the same unit (eg, kilos). The third stage is calculating similarity measures. The similarity measure indicates the strength of the relationship between two objects. In our case, each object refers to an elderly patient.

One of the main features of clustering is the calculation of a degree of similarity between individuals. There are several methods to calculate matrices of similarity, dissimilarity, and distance [22] among individuals in a population. At this time, Gower General Similarity Coefficient is one of the most popular measures of proximity or similarity when there are variables with different data types [23]. The Gower coefficient allows the determination of the degree of similarity between 2 individuals or cases (*i, j*) that present binary, qualitative, and quantitative data. Other algorithms do not allow these advantages. In Multimedia Appendix 2, mathematical formulas to calculate the similarity values based on the Gower coefficient are described in detail.

In our case, calculating similarity coefficients from frailty factors involves working with mixed variables (qualitative, quantitative, and binary) as we mentioned above, and even creating diagnostics depending on the situation; thus, the application of the Gower algorithm is quite suitable in this context. Through the use of this coefficient, it is possible to weight the frailty variables independently, depending on the importance the doctor determines in the moment. Therefore, physicians could perform a frailty assessment focusing on specific areas, such as physical, nutritional, cognitive, and anthropometric among others.

**Table 2:** Inputs and outputs of identified services (services are mobile or Web, depending on the running device, and their outputs are typically the inputs of the next service).

Service	Type	Description	Inputs	Outputs
Accelerometer data acquisition	Mobile	Responsible for accelerometer data gathering and storage at run time, when elderly people perform a specific gait and balance test. This also includes mobile communication between the smartphone and the accelerometer sensor.	Accelerometer signal	Accelerometer values in x,y,z axes
Accelerometer data processing	Mobile	Responsible for accelerometer data handling through data filtering and segmentation as well as calculation of accelerometry indicators.	Accelerometer values (x,y,z axes)	Accelerometry indicators (dispersion measures)
Patient record extraction	Web	Defines the mechanisms to obtain frailty risk factors from the patient record. The use of clinical standards could be necessary.	Patient record, accelerometry indicators	Frailty risk factors
Frailty study procedure	Web	Responsible for performing a comparison between frailty risk factors from the elderly patient studied and each of the patients stored in the database (known as patient stack).	Frailty risk factors, patient stack	Frailty assessment
Setting up a built result	Web	Parse the comparison results in a formal language, easily readable by the mobile phone.	Frailty assessment	Frailty assessment formalized
Visualization of frailty assessment	Mobile	Defines the method for frailty result preparation and visualization on the smartphone screen, after receiving data from the server.	Frailty assessment formalized	Information, tips, and charts for the physician
Storage into Patient Stack	Web	Stores the new patient data in the patient stack structure, increasing the patient stack size and improving the accuracy of frailty assessments in the future.	Risk factors from a new patient	Patient stack with new patient

## Results

### Summary

**Figure 2:**
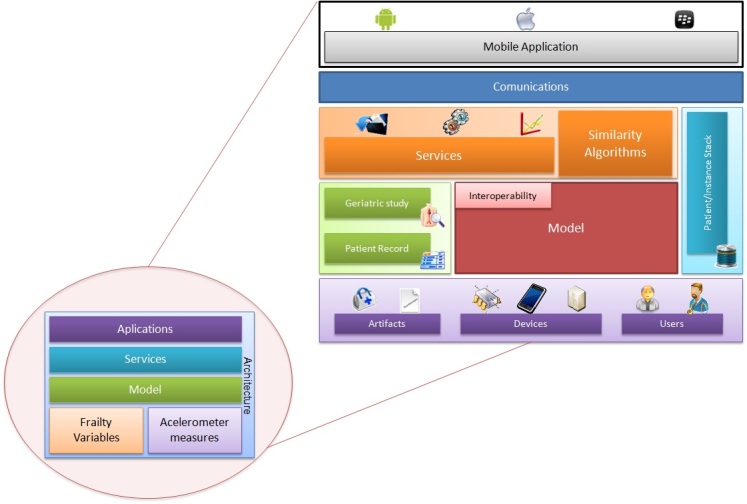
General overview of the architecture of the developed system.

Figure 2 shows an overview of the developed system according to the specifications described in this paper. The model integrates all components and allows for the development of mobile applications to determine the final frailty assessment. The architecture of this system has been divided into layers and blocks corresponding to each described part. In this sense, the model ensures the interoperability between the rest of the elements, leading to the services that are used on the mobile phone for data acquisition, processing, and visualization of frailty results.

**Figure 3:**
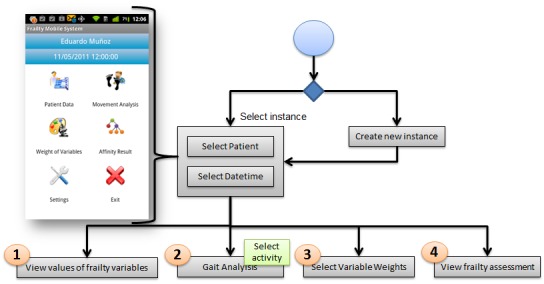
Flow diagram and screen capture of the application dashboard.

**Figure 4:**
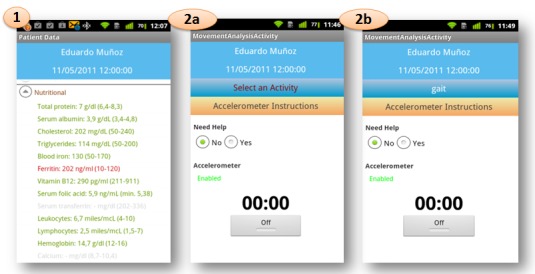
Screenshots from functionalities from mobile application flow: values of the frailty variables for a specific patient; movement analysis task before the activity selection and the start for a specific patient; and movement analysis task after the activity selection (gait) and before the start for a specific patient.

**Figure 5:**
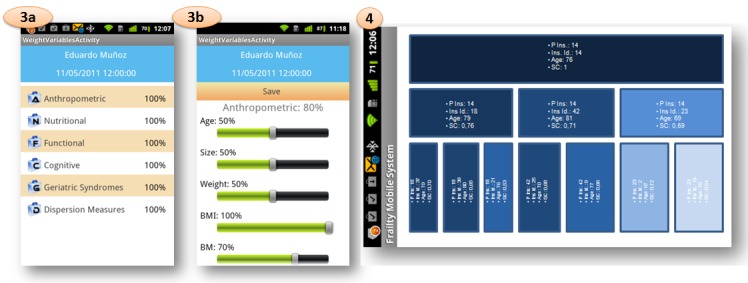
Screenshots from functionalities from mobile application flow: total weight of each group of frailty variables for a specific patient; editing of frailty variables weight for a specific group of variables and patient; and example of treemap calculated for a specific patient.

In this approach, a mobile application has been developed to provide a frailty assessment for each new incoming elderly patient by considering data from a group of previously studied elderly people. This application allows us to display the values of every frailty variable from the patient record, extract dispersion measures from a gait exercise, adjust the variable weights, and visualize the similarity results. Figure 3 shows the mobile application flow with the options to be chosen by the user, and Figures 4 and 5 show screen captures corresponding to these options.

The results are presented in a treemap view (see Figure 5), providing the physician with a visual as support for making the final diagnosis for the selected patient. In our case, this treemap consists of nodes, where each node (represented by a rectangle) is a dynamic object through which the user can access the full information of the patient instance represented by that particular node, including all values of frailty variables. Specifically, a node object contains the following attributes: Parent Instance ID, corresponding to the patient instance identifier of the parent node; Instance ID, referring to the identifier of the current patient instance (the instance to be studied) associated with the node; Age, referring to the age of the patient represented by that node; and Similarity Coefficient, showing the value of the similarity coefficient between the parent node and the current node.

Obviously, if there are large numbers of patient instances, the system will need more processing capacity, more storage resources, better memory management, and more time to generate the similarity results. However, in addition to the optimization of these system features, we propose reductions in the depth of the treemap (to a maximum of three levels) and the maximum number of child nodes for each parent node (to three). These recommendations are suggested because with greater numbers of tree levels and child nodes, the frailty assessment service would require greater processing time (depending on the stored patient instances), and the final results would also not be as useful to the physician (because the similarity coefficient values are lower at each tree level in relation to the studied instance, corresponding to the root node). In our case, the maximum time to generate a complete treemap on the mobile screen was 2.55 seconds working on 64 instances from 20 different patients (see next section). Although four node lists are calculated (for the root node and the three children of the second tree level), only the most representative nodes are shown depending on their similarity coefficients.

### Study 1: Frailty Evaluation

The system was evaluated on 20 elderly patients with an average age of 83.58 years (SD 3.98), including 10 women (mean age 85.43, SD 3.22) and 10 men (mean age 81.80, SD 4.74). Information on each patient was collected on three occasions over a period of 1 year. The collected information included all possible values of frailty variables from the patient record and those related to gait exercise. In Tables 3 and 4, we present all studied instances considering six different domains (see “Identification of Frailty Factors” section): Anthropometric (with 9 variables), Functional (with 6 variables), Nutritional (13 variables), Cognitive (2 variables), Geriatric syndromes (11 variables), and Dispersion measures (20 variables).

Considering all the previous variables for each group (see table headings of Tables 3 and 4), we observed that there were some variables without value for two different reasons. First, when a variable was measured and its value was in range (within the normal clinical bounds), dependent variables of this were not measured (eg, “if total protein is in a normal range, serum albumin could not be given”); this consists of a clinical decision. And second, certain values simply do not appear. Table 5 shows a summary of the total number of existing values from Tables 3 and 4.

According to the values associated with the variables, the first iteration (from instances 1 to 20) presents more given values. Thus, we selected this iteration as the representative example for frailty assessment because more values imply more accuracy. Suppose the frailty assessment is made from instance 1 (with the maximum of importance for all variables: 100%). This instance is compared, through the similarity process, with the other 19 instances. Then, a list of nodes, based on their similarity coefficients, is calculated from the current instance. This process is repeated for each child node (with a maximum of three nodes per tree level and two levels). Multimedia Appendix 3 presents these results, which are formalized in a treemap structure, their order by their similarity coefficients, and displayable on a mobile device.

In this case, instance 1 has a similarity degree of 73.4% with instance 12 (corresponding to Patient 12), 72% with instance 16, and 71.6% with instance 2. In the same way, similarity coefficients from these last instances were calculated. The instances of the last node present lower similarity values, as calculated from their parent nodes. In this case, the third node of the second level (with instance ID=2, corresponding to Patient 2) has the worst similarity coefficient, and its child nodes present similarity degrees of 71.9%, 71.6%, and 69.9%. This indicates lower degrees of similarity in relation to the rest of the nodes. This case can be extrapolated to any other generated tree. For the geriatrician, calculating more child nodes would result in a confusing interpretation with useless results.

However, taking into account the whole group of instances, those from the generated result correspond to patients within limited age ranges. In this case, no result shows similarity between the studied instance and those patients less than 80 years or greater than 90 years. In our experiment, we had a 92-year-old woman and 4 individuals aged less than 80 years. This indicates the importance of setting clusters based on sex and age before conducting the similarity calculations, optimizing the results of the system (which does not currently offer this feature).

These results help the physician to determine the frailty condition of a specific patient in relation to other patients in an elderly population. Additionally, from these results, the doctor can group the elders by their degree of similarity influencing their care and treatment.

**Table 3:** Values of frailty variables (anthropometric, functional, and nutritional) for all studied patient instances; the first iteration presents more variables with existing values (k=value kept, 0=values not recorded).

Patient	Sex	Instance ID			Anthropometric (max. 9)			Functional (max. 6)			Nutritional (max. 13)		
1	M	1	22	47	9	8	8	4	k	0	11	9	8
2	M	2	23	48	9	8	8	5	k	0	11	8	8
3	M	3	24	49	9	8	8	4	k	0	0	7	0
4	M	4	25	50	9	8	8	5	k	0	12	8	8
5	M	5	26	51	8	8	8	4	k	0	11	8	9
6	F	6	27	-	9	8	-	4	k	-	11	10	-
7	F	7	28	52	8	8	8	5	k	0	12	7	8
8	F	8	29	53	8	8	8	4	k	0	12	9	8
9	F	9	30	54	9	8	8	4	k	0	12	8	8
10	F	10	31	55	8	8	8	5	k	0	12	8	8
11	F	11	32	56	9	8	8	4	k	0	11	10	9
12	F	12	33	57	9	8	8	4	k	0	11	10	9
13	F	13	34	58	9	8	8	4	k	0	11	9	9
14	F	14	35	-	2	8	-	4	k	-	10	10	-
15	F	15	36	59	9	8	8	4	k	0	11	12	10
16	M	16	37	60	9	8	8	4	k	0	12	10	10
17	M	17	38	61	9	8	8	4	k	0	12	10	9
18	M	18	39	62	9	8	8	4	k	0	12	9	10
19	M	19	40	63	9	8	8	4	k	0	12	9	9
20	M	20	41	64	9	8	8	4	k	0	11	9	9

**Table 4:** Values of frailty variables (cognitive, getriatric syndromes, and dispersion measures) for all studied patient instances; first iteration presents more variables with existing values (k=value kept, 0=values not recorded).

Patient	Sex	Instance ID (It. 1/It. 2/t. 3)			Cognitive (max. 2)			Geriatric syndromes (max. 11)			Dispersion measures (max. 20)		
1	M	1	22	47	2	0	0	11	k	k	17	0	20
2	M	2	23	48	1	0	0	11	k	k	17	0	20
3	M	3	24	49	0	0	0	11	k	k	17	0	20
4	M	4	25	50	1	0	0	11	k	k	17	0	20
5	M	5	26	51	0	0	0	11	k	k	17	0	20
6	F	6	27	-	1	0	0	11	-	k	17	0	-
7	F	7	28	52	1	0	0	11	k	k	17	0	20
8	F	8	29	53	1	0	0	11	k	k	17	0	20
9	F	9	30	54	0	0	0	11	k	k	17	0	20
10	F	10	31	55	1	0	0	11	k	k	17	0	20
11	F	11	32	56	2	0	0	11	k	k	17	0	20
12	F	12	33	57	2	0	0	11	k	k	17	0	20
13	F	13	34	58	2	0	0	11	k	k	17	0	20
14	F	14	35	-	2	0	0	11	k	-	17	0	-
15	F	15	36	59	2	0	0	11	k	k	17	0	20
16	M	16	37	60	2	0	0	11	k	k	17	0	20
17	M	17	38	61	2	0	0	11	k	k	17	0	20
18	M	18	39	62	2	0	0	11	k	k	17	0	20
19	M	19	40	63	2	0	0	11	k	k	17	0	20
20	M	20	41	64	1	0	0	11	k	k	17	0	20

**Table 5:** Existing values (this indicates the total number of variables for each iteration item with a value).

Existing values	
1^st^ iteration (instances 1-20)	1057
2^nd^ iteration (instances 22-41)	644
3^rd^ iteration (instances 47-64)	913

### Study 2: Evolution in Frail Elderly Focusing on Nutritional Aspects

In a second study, the mobile application for frailty assessment was used to perform an evaluation on 10 elderly patients (5 men and 5 women) with nutritional deficiencies, from the initial group of 20 elderly patients, according to the criteria of the geriatrician. In this case, we consider only some frailty variables from the nutritional and anthropometric domains, specifically the following: weight, body mass index, fat mass, lean mass, total water, total protein, hemoglobin, serum albumin, and lymphocytes. The weight of these variables was set to 1 (100% of importance) in the mobile application, and the weights of the remainder variables were set to 0 (0% of importance).

The following three stages were established to assess each of the elders. Stage 1 is the Initial assessment, referring to the acquisition of the previous frailty parameters from the first instance of each patient stored in the system. Stage 2 is Spontaneous evolution, corresponding to the second assessment of the whole group of the parameters considered for the patients. After 9 months, a second instance of the patients was created and the values of their frailty variables were studied. Finally, Stage 3 is Assessment after protein supplementation, referring to the last assessment of the group of elderly patients. In this case, the elderly patients had taken 220 mL, twice a day, of a protein supplement for 2 months. After that, a third instance of the patients was created and the values from the frailty variables of the new instances were analyzed again.

From Stage 1, we made a general description of the group. We observed a higher weight average in males and higher values of lean mass and total water. However, females had higher values of body mass index and fat mass. According to the standard limits for the body mass index determined for the World Health Organization, 2 males and 1 female were at risk of malnutrition. Additionally, values of total protein and lymphocytes were too low. Table 6, Stage 1, shows the average of the values of studied variables, for men and women, initially. Table 6, Stages 2 and 3 show the corresponding values for the same variables in the spontaneous evolution assessment and the assessment after protein supplementation.

From Stage 1 to Stage 2, most values decrease in both men and women. It indicates an increase in the frailty state of the elderly patients. Additionally, in the clinical spontaneous evolution, the geriatrician determined that the biochemical parameters were affected earlier than the anthropometric.

**Figure 6:**
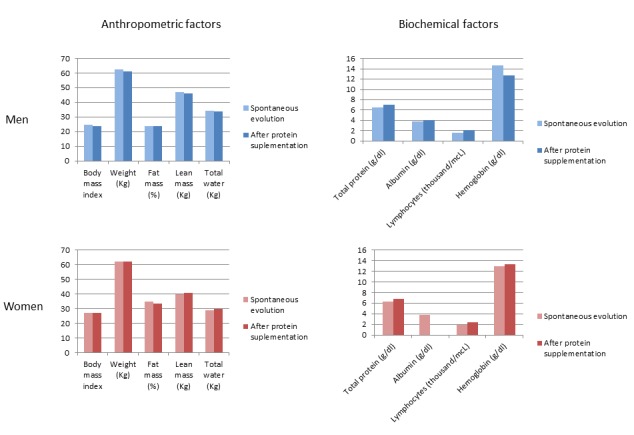
Values of the selected frailty variables for men and women in the stages: spontaneous evolution and assessment after protein supplementation.

On the other hand, from Stage 2 to Stage 3, most variables maintain their values, and even some of these values are increased. This is due to the supplementation. In this case, women present a greater increase in the values of more variables than men. Figure 6 shows several charts regarding the values of the frailty variables in men and women, addressing Stages 2 and 3.

In this study, the frailty mobile application has been useful for performing an evolutionary analysis from a nutritional viewpoint, taking into account a subset of variables (modifying their weights from the application). Additionally, we observed the improvement of biochemical and anthropometric values after supplementation. In men, values from biochemical variables were improved. In women, values from anthropometric and biochemical domains were increased, observing that, from a nutritional viewpoint, the administration of protein supplements may help delay frailty in certain cases.

**Table 6:** Evolution of average values of selected frailty factors for the group of 5 men and 5 women (previously selected).

		Anthropometric					Nutritional			
	Sex	Body mass index	Weight (kg)	Fat mass (%)	Lean mass (kg)	Total water (kg)	Total protein (g/dl)	Albumin (g/dl)	Lymphocytes (thousand/mcl)	Hemoglobin (g/dl)
**Stage 1: Initial assessment**										
	Male	26.19	67.78	27.61	48.29	35.35	6.88	3.95	1.9	14.57
	Female	28.14	65.45	36.37	40.93	31.11	6.95	4.18	1.71	12.92
**Stage 2: Spontaneous evolution**										
	Male	24.58	62.42	23.99	47.23	34.58	6.48	3.83	1.58	14.64
	Female	27.2	62.2	35	39.68	29.04	6.3	3.83	1.86	13
**Stage 3: Assessment after protein supplementation**										
	Male	23.97	61.38	23.87	46.35	33.93	6.97	4	2.08	12.76
	Female	27.28	62.38	33.37	40.86	29.92	6.86	No data^a^	2.36	13.36

^a^Not enough data to calculate the average.

## Discussion

### Principal Findings

The creation of an absolute index that determines the frailty condition of an elderly patient is still an unsolved issue. However, several proposals provide approximations toward this goal, as we have seen in some related works. In this work, we presented a model with several features to develop mobile tools for frailty assessment. Additionally, we implemented a mobile system based on this model to support elderly frailty diagnosis in a health care environment. The proposed system consists of a model that defines all the needed elements, relationships, and functionalities (using a service-oriented approach) for frailty assessment.

Nowadays, many physicians emphasize the lack of a centralized method to provide frailty assessments based on the results of existing tests and clinical information. For this purpose, we can take advantage of mobile device features such as accelerometer sensors, wireless communication capabilities, and processing capacities, among others, to develop new methods and mechanisms that lead toward an objective assessment of frailty. Additionally, this system can be deployed on other devices, especially for better results visualization (eg, widescreens); however, the mobile device is also used for this task due to the heterogeneity and mobility of the clinical environments, getting evaluations at any time (when the physician needs them).

In a complementary fashion, we consider the use of similarity algorithms, which take into account all relevant frailty variables from the patient record to support the clinical decisions regarding the frailty state of an elderly person in comparison to an elderly population. In this case, due to the nature of the studied variables (quantitative, qualitative, and binary), the Gower algorithm provides us with the most appropriate method to obtain similarity values from a group of patient instances. Moreover, the obtained results are transformed into objects called nodes, which are represented in a treemap structure according to the similarity values of each node. With more patient instances and existing values related to frailty variables, the system results will increase accuracy and reliability.

The lack of similar systems in the literature means that we cannot compare our proposal with other systems. However, the development of mobile computing in the health care domain and the interest in frailty studies because of the growth of the elderly population in developed countries are the main reasons why new systems with similar approaches are being developed.

In the two studies conducted, we checked the usefulness of the mobile application for supporting the frailty diagnosis as well as other kinds of related studies. In this case, we have also presented the use of the mobile application to perform an evolutionary analysis based on nutritional parameters and a subset of elderly patients.

### Limitations

Finally, we can further evaluate the integration of clustering techniques in our system as a complement to a thorough study, taking into account different populations of adults and elderly patients. For this, it is necessary to have a large group of patient instances with a wide range of ages. Although this feature can be applied to our evaluation group of elders, results would not be reliable because the sample is too small and it is difficult to establish generalized cutoffs based on ages. Additionally, possible optimizations related to processing and performance may be necessary to handle the corresponding large amounts of data.

## Multimedia Appendix 1

Model entities and roles to develop frailty assessment tools.

## Multimedia Appendix 2

Gower's Coefficient equation.

## Multimedia Appendix 3

Values of the treemap nodes in table format.
