# Supplementary material for: A Mobile and Ubiquitous Approach for Supporting Frailty Assessment in Elderly People
Source: J Med Internet Res. 2013 Sep 4;15(9):e197. doi: 10.2196/jmir.2529 (PMC3785993; doi:10.2196/jmir.2529)
Supplement: Supplementary file 1 [file jmir_v15i9e197_app1.pdf]

Multimedia Appendix 1: Model entities and roles to develop frailty assessment tools.

Table 1. Model entities and roles. In the proposed model, each entity belongs to a specific class and has one or more associated roles[1].

Class	Entity	Role
Device	Mobile phone	Service deployment, interaction facilitation, visualization, accelerometer data acquisition
	Accelerometer	Accelerometer data acquisition (in special purpose devices)
	Server	Service requests from mobile phone, process deployment
User	Elderly patient	Carry mobile phone / accelerometer on the waist during physical activity, provide the patient record
	Physician	Assess frailty state of the elder, interact with mobile phone
Artifact	Questionnaires	Provide one or more measures for a general assessment based on clinical indicators from the physician's viewpoint (e.g., Barthel Index test)
	Medical instrument	Provide accurate measures of clinical indicators from clinical equipment (e.g., Body Mass Index scales)
Procedure	Patient record	Provide values of risk factors for frailty
	Patient stack	Frailty data storage from previously studied patients
Service	Acquisition	Acquisition services get data from the corresponding entities, including accelerometer data, patient record data and patient stack data
	Processing	Analysis and processing of accelerometer data, patient record data and patient stack information
	Storage	Data storage used by entities and processing tasks
	Results	Create a structure for visualization of frailty results on mobile phone screen

The Procedure and Service entities correspond to software components, which must be previously implemented unlike tangible entities (devices, users and artifacts).

[1] Fontecha J, Hervás R, Bravo J, Navarro FJ. A model to develop frailty diagnosis tools through mobile devices and service-oriented approach. *Ambient Assisted Living and Home Care*, Springer LNCS, 2012;7657:375-382. ISBN:978-3-64235394-9

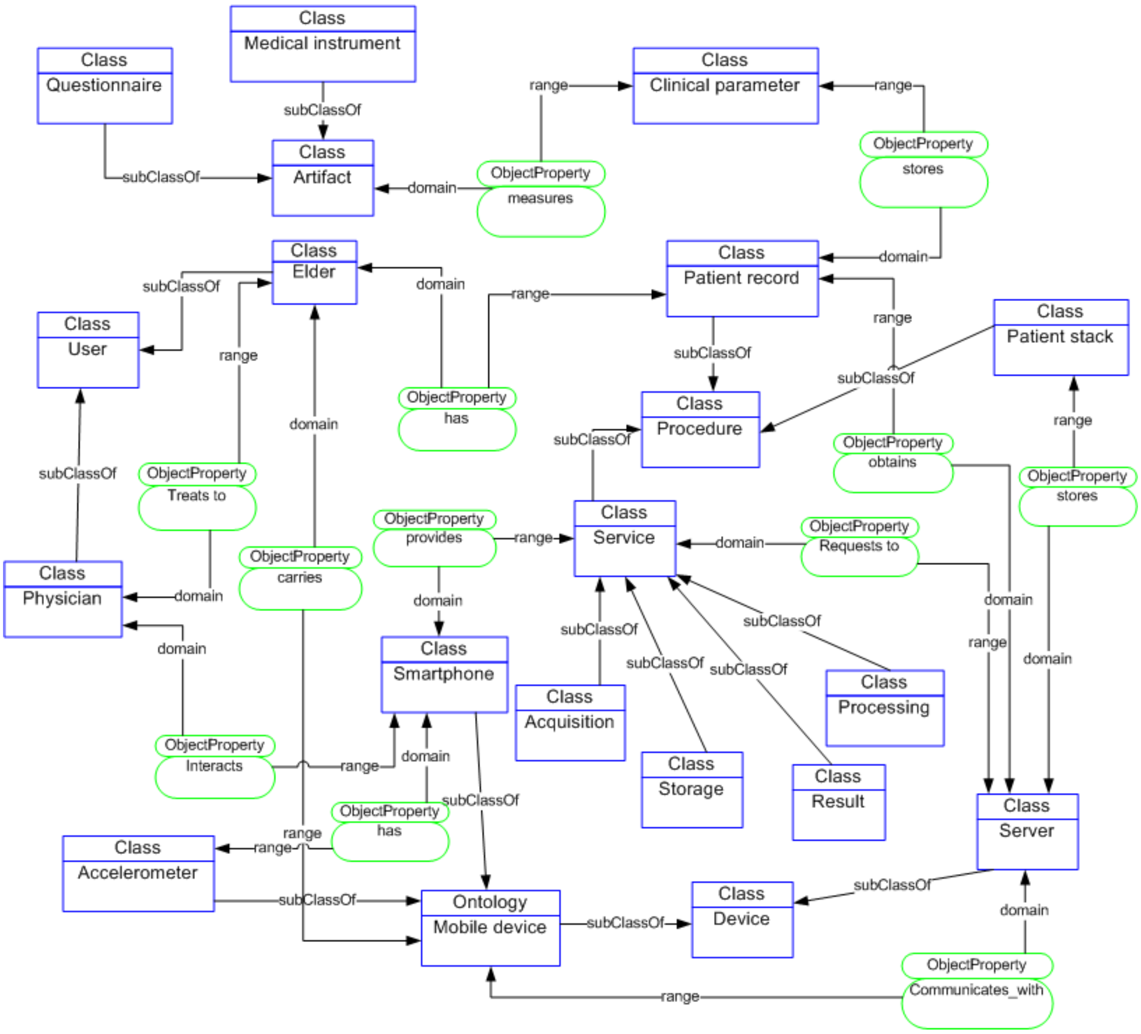

Figure 1. Graphical representation of the model structure

The previous entities are all related to one another. For additional clarification, Figure 1 shows a graphical representation of the model according to Table 1, in which the relations between entities represent the model assumptions; these are defined as follows:

- Physician-*Treats*-Elder. Physician is responsible for treating an elder.
- Elder-*has*-Patient record. The elder has a patient record.
- Elder-*carries*-Mobile device. The elder carries an accelerometer-enabled mobile device during gait and balance tests.

- Physician-*interacts with*-Smartphone. The physician interacts with the smartphone to know the patient state.
- Smartphone-*has*-Accelerometer. The accelerometer can be integrated into the smartphone.
- Smartphone-*provides*-Service. All identified services (acquisition, storage, results and processing) are provided by the smartphone.
- Service-*Requested of*-Server. The service is requested of the server.
- Server-*Communicates with*-Mobile device. The server sends the corresponding result to the smartphone.
- Server-*obtains*-Patient record. The server obtains the patient record as a medical resource.
- Server-*stores*-Patient stack. The server stores the patient stack entity.
- Patient record-*stores*-Clinical parameters. Additionally, the patient record stores a set of clinical frailty parameters.
- Artifact-measures-Clinical parameters. An artifact (questionnaires and medical instruments) measures a clinical parameter.
