# Supplementary material for: A Mobile and Ubiquitous Approach for Supporting Frailty Assessment in Elderly People
Source: J Med Internet Res. 2013 Sep 4;15(9):e197. doi: 10.2196/jmir.2529 (PMC3785993; doi:10.2196/jmir.2529)
Supplement: Supplementary file 2 [file jmir_v15i9e197_app2.pdf]

Multimedia Appendix 2: Gower's Coefficient equation.

Equation 1 defines Gower's Coefficient:

$$S_{ij} = \frac{\sum_{k=1}^{p} W_{ijk} S_{ijk}}{\sum_{k=1}^{p} W_{ijk}} \quad (1)$$

where:

- S_{ijk} denotes the contribution provided by the *k*th variable,
- W_{ijk} is usually 1 or 0 depending upon whether or not the comparison is valid for the *k*th variable; if differential variable weights are specified it is the weight of the *k*th variable, or it is 0 if the comparison is not valid,
- the effect of the denominator is to divide the sum of the similarity scores by the number of variables; or if variable weights have been specified, the sum of their weights.

Gower defines the value of S_{ijk} for quantitative variables as shown in Equation 2:

$$S_{ijk} = 1 - \frac{|x_{ik} - x_{jk}|}{R_k} \quad (2)$$

where:

- R_k is the range of values for the *k*th variable, for quantitative variables, S_{ijk} range is between 0 and 1, applying the formula $|x_{max} - x_{min}|$.

For binary and qualitative data, we consider that S_{ijk}=1, if x_{ik}=x_{jk}, or S_{ijk}=0, if $x_{ik} \neq x_{jk}$.

It was noted above that the weight W_{ijk} for the comparison of the *k*th variable is usually 1 or 0. However, if we assign differential weights to the variables, then W_{ijk} is either the weight of the *k*th variable or 0, depending upon whether the comparison is or is not valid. This allows for considering the particular importance of each variable (given by weight) in the similarity calculation. If the weight of any variable is 0, then the variable is effectively ignored for the calculation of proximities or similarities. Such variables are "masked" for clustering and classification, but these may be taken into account to assist in the interpretation of a resulting cluster analysis.
