# Supplementary material for: A Mobile and Ubiquitous Approach for Supporting Frailty Assessment in Elderly People
Source: J Med Internet Res. 2013 Sep 4;15(9):e197. doi: 10.2196/jmir.2529 (PMC3785993; doi:10.2196/jmir.2529)
Supplement: Supplementary file 3 [file jmir_v15i9e197_app3.pdf]

Multimedia Appendix 3: Values of the treemap nodes in table format

Table 1. Results of frailty assessment related to patient instance 1

**Level 1 (root)**									
Parent instance	1								
Instance Id.	1								
Name	Patient 1								
Age	81								
Similarity coeff.	1								
**Level 2**									
Parent instance	1			1			1		
Instance Id.	12			16			2		
Name	Patient 12			Patient 16			Patient 2		
Age	85			87			88		
Similarity coeff.	0.734			0.72			0.716		
**Level 3**									
Parent instance	12	12	12	16	16	16	2	2	2
Instance Id.	15	16	1	18	12	1	16	1	20
Name	P15	P16	P1	P18	P12	P1	P16	P1	P20
Age	81	87	81	87	85	81	87	81	85
Similarity coeff.	0.751	0.75	0.734	0.773	0.75	0.72	0.719	0.716	0.699

The nodes of the final treemap are ordered by their similarity coefficient as shown in the table.
